# Potential Causes of Shedding Aggregations in Prairie Rattlesnakes

**DOI:** 10.1002/ece3.73311

**Published:** 2026-03-26

**Authors:** Emily Martin, Courtney J. Conway

**Affiliations:** ^1^ Idaho Cooperative Fish and Wildlife Research Unit University of Idaho Moscow Idaho USA; ^2^ US Geological Survey, Idaho Cooperative Fish and Wildlife Research Unit Moscow Idaho USA

**Keywords:** aggregation, behavior, *Crotalus viridis*, ecdysis, snake

## Abstract

Aggregation is common across taxa and typically confers clear benefits to group members (e.g., allo‐parenting, group defense, thermoregulation, access to resources). But aggregation can also be costly. The mechanisms that underpin aggregation—and the cues that elicit it—inform our understanding of how animals resolve tradeoffs among selection pressures. Snakes sometimes form conspicuous aggregations associated with hibernation, gestation, or parturition. Aggregation during ecdysis has also been described in some species, but infrequent observations and the synchronicity of ecdysis have confounded attempts to deduce the mechanism(s) responsible for the behavior. We documented aggregation during ecdysis in a population of asynchronously shedding prairie rattlesnakes ( *Crotalus viridis* ) and tested predictions generated from four hypotheses proposed to explain this behavior. We found that individuals undergoing ecdysis were more likely to aggregate. Our data did not support the hypothesis that rattlesnakes aggregate to improve their thermal efficiency, but we found some support for the reproductive facilitation and thermal landscape hypotheses as possible explanations for aggregation during ecdysis.

## Graphical abstract

The mechanisms that underpin aggregation behavior in animals—and the cues that elicit it—inform our understanding of how animals resolve tradeoffs among selection pressures. We documented aggregation during ecdysis in a population of asynchronously shedding prairie rattlesnakes ( *Crotalus viridis* ) and tested predictions generated from the four hypotheses proposed to explain this behavior.

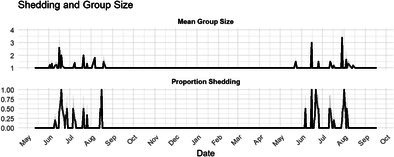

## 1 Introduction

Squamate skin is an adaptation to life on land and a key aspect of the persistence of modern reptiles (Alibardi 2003). The highly keratinized outer epidermis is a mechanical barrier to injury and pathogens, while inner layers regulate cutaneous water loss and generate new cells (Wagner et al. 2023). The limited elasticity of squamate skin prevents it from expanding continuously as individuals grow or repair damage (Maderson 1965). To overcome this hurdle, squamates undergo periodic ecdysis: a hormone‐regulated process where they shed and synchronously replace their entire epidermis (Alibardi 2003). Ecdysis may play a role in hormone signaling or enhancing chemical communication (Carnes‐Mason 2023), but it also imposes substantial energetic demands. Skin biosynthesis and removal can comprise 3%–11% of an individual's annual energy budget (Carnes‐Mason et al. 2024; Wagner et al. 2023). Furthermore, increased metabolic demand and a disrupted epidermal barrier during ecdysis increase cutaneous water loss (33% in *Vipera aspis* ), which can affect thermoregulation (Dupoué et al. 2015). Ecdysis can also make animals more vulnerable to predation (Wagner et al. 2023). Clouding of the visual spectacle impairs predator detection, while skin sloughing disrupts chemical crypsis and camouflage (Miller et al. 2015).

Free‐ranging animals can offset some of the physiological tradeoffs imposed by ecdysis by modifying their behavior (Wagner et al. 2023). Some individuals prioritize thermoregulation and promote new epidermal growth by raising their internal body temperature (Brown et al. 1982). Others prioritize predator evasion by becoming reclusive and reducing time spent foraging or basking (Kitchell 1969). Understanding the mechanisms that underlie behavioral changes during ecdysis can be informative (1) as a method to infer the processes governing selection and (2) to determine the role of behavioral plasticity in navigating trade‐offs.

Some behavioral changes that occur during ecdysis—such as aggregation—are difficult to observe or have not been linked to a causal mechanism. Aggregation typically confers clear selective advantages to group members, such as increased thermal efficiency, risk mitigation, and enhanced breeding opportunities (Chapperon and Seuront 2012; Lehtonen and Jaatinen 2016; Brockmann et al. 2018). However, aggregation behavior also has potential costs, such as predator attraction, heightened intraspecific competition, or disease transmission (Allee 1927). Given these tradeoffs, understanding how and why animals aggregate provides insight into the processes that shape community dynamics, co‐existence, life history strategies, and species persistence (Wertheim 2005).

Synchronized, aggregated ecdysis appears in the fossil record as early as the Cambrian period and persists in some modern arthropods (Haug et al. 2013; Kim 2001). Aggregation during ecdysis has also been documented in several species of modern snake, including several species of rattlesnake (Loughran et al. 2015; Ashton 1999). Snakes often form cue or self‐organized aggregations (Noble and Clausen 1936), as well as aggregations around heterogeneously distributed resources (Graves and Duvall 1995; Gregory 2004). Rattlesnakes (genera *Crotalus* and *Sistrurus*) sometimes form large seasonal aggregations that coincide with hibernation (including the period preceding ingress and following egress), gestation, or parturition (Klauber 1956; Graves and Duvall 1993, 1995; Clark et al. 2014). Some rattlesnakes also form smaller, ephemeral breeding aggregations, which are the result of multiple males converging on a receptive female (Schuett et al. 2002, 2016). Within aggregations, individuals may exhibit nuanced social behavior, compete for resources, and engage in group defense (Matsumoto and Mori 2024; Hoss and Clark 2014). The characteristics of these aggregations provide insight into the selective advantages to aggregation in these contexts, and rattlesnake aggregations outside of these contexts are rare (or rarely reported). However, multiple shed skins and groups of shedding rattlesnakes have been reported throughout the year—observations that suggest that some rattlesnakes also aggregate during ecdysis (Loughran et al. 2015). Shedding aggregations have been observed in synchronously and asynchronously shedding populations, and the timing, sex composition, and behavior within shedding aggregations vary (Loughran et al. 2015; Parker and Anderson 2007).

In a Washington population of *Crotalus oreganus*, shedding aggregations contained an estimated 2–5 individuals and were detected during the latter half of the active season (July–September). Copulation was sometimes witnessed, though not all aggregations contained both sexes. Pregnant females were more common than nonpregnant females, and shedding locations were re‐used between and within active seasons (Loughran et al. 2015). Shedding aggregations of *C. concolor* in Wyoming—where ecdysis is more synchronized—also contained an estimated 2–5 individuals but were detected earlier in the active season (May–July). Copulation was not witnessed within shedding aggregations and this population likely mates in late summer. Only males and pregnant females were detected within aggregations, and shedding locations were re‐used between active seasons (Parker and Anderson 2007).

Though the cause(s) of aggregation during ecdysis remain unknown, several hypotheses have been proposed to explain this behavior.

### 1.1 Thermal Landscape

Rattlesnakes may select for higher internal body temperatures during ecdysis to accommodate increased metabolic demand or speed cellular growth (Hoffman et al. 2021). If suitable microhabitats are limited, rattlesnakes may aggregate during ecdysis in locations that allow them to optimize thermoregulation (Graves and Duvall 1987). The thermal landscape hypothesis makes several predictions about timing and composition of these aggregations:

1. Thermal quality of rattlesnake habitat varies across multiple temporal scales (Rowe et al. 2022). Seasonal variation places the highest thermal demands on rattlesnakes at the beginning and end of the active season (May, June, and September in *Nerodia sipedon*, e.g., Rowe et al. 2022). Thus, the thermal landscape hypothesis predicts that rattlesnake aggregations will be most common during and immediately following egress, as well as immediately preceding ingress—when the benefits of aggregation are highest.
2. Pregnant female rattlesnakes may be more constrained to habitats with higher thermal stability (Moniz et al. 2024). Therefore, the thermal landscape hypothesis predicts that pregnant females should be more likely to be found within aggregations.
3. Juvenile rattlesnakes may select from a wider variety of temperatures along a thermal gradient than adults and spend less time on the surface to avoid predators (Loughran et al. 2025). Therefore, the thermal landscape hypothesis predicts that juveniles should aggregate less frequently than adults.

### 1.2 Thermal Efficiency

Aggregated snakes have higher thermal inertia than solitary snakes (Graves and Duvall 1987), and rattlesnakes may be more sensitive to thermal variation during ecdysis (Hoffman et al. 2021). By aggregating during ecdysis, rattlesnakes could buffer against thermal instability. The predictions for the thermal efficiency hypothesis are:

1. As with the thermal landscape hypothesis, the thermal efficiency hypothesis predicts that aggregations will be more common during ingress and egress, rather than associated with ecdysis per se.
2. Pregnant female rattlesnakes prioritize thermal stability during gestation (Moniz et al. 2024). Thus, the thermal efficiency hypothesis predicts that pregnant female rattlesnakes will be more likely to aggregate than males or nonpregnant females.
3. Smaller, juvenile rattlesnakes experience higher cooling rates and have lower thermal inertia than adults (Pearson et al. 2003). In contrast to the thermal landscape hypothesis, the thermal efficiency hypothesis predicts that juveniles will be more likely to aggregate than adults.

### 1.3 Predator Avoidance

During the later stages of ecdysis, snakes experience impaired visual acuity and reduced overall activity (King and Turmo 1997). Rattlesnakes may use aggregation to increase visual detection of predators or to reduce individual predation risk (Lehtonen and Jaatinen 2016). The predator avoidance hypothesis predicts:

1. Aggregations will be associated with ecdysis only, rather than with ingress or egress.
2. Behavioral differences among sex classes can affect predation of some snakes (Olson et al. 2015; Noble et al. 2025), but the directionality of this relationship appears situational in rattlesnakes. Therefore, the predator avoidance hypothesis makes no strong prediction about the sex classes that should be present within aggregations.
3. Size‐ or age‐dependent predation risk has not been reported in snakes, though mortality is generally higher in juveniles than adults (Brown et al. 2007). Therefore, the predator avoidance hypothesis makes no strong prediction about the age classes that should be present within aggregations.

### 1.4 Reproductive Facilitation

Positive temporal associations between ecdysis and reproduction have been documented in some rattlesnakes. Male *C. oreganus* often court and mate within 48 h of ecdysis (Macartney and Gregory 1988) and, in some species, females undergo a “pre‐mating” shed that precedes courtship (Brown 1995). Aggregation during and immediately following ecdysis could facilitate mate‐guarding by males or permit males to capitalize on a short breeding window (Duvall et al. 1992). The reproductive facilitation hypothesis predicts:

1. Rattlesnakes should aggregate only during mating.
2. Female rattlesnakes are capable of long‐term sperm storage (up to 6 years in *C. atrox*, Levine et al. 2021), and pregnant females may still be found in breeding aggregations even if they are unlikely to reproduce the following year (Lind et al. 2016). Thus, the reproductive facilitation hypothesis predicts that aggregations should contain all sexes, but at least one male and one female in each.
3. Juveniles—which are not capable of reproduction—should not aggregate.

Differentiating among these 4 causal hypotheses could provide insight into the processes that govern habitat use, reproduction, and movement, and could also improve detection of Allee effects in species of conservation concern (Allee 1927; Kramer et al. 2009). However, in some populations that form shedding aggregations, ecdysis is synchronous and occurs approximately the same time each year (Parker and Anderson 2007). This synchronicity amplifies the confounding effects of other temporally mediated life history events (ex: reproduction, hibernation) and complicates our ability to test predictions. Populations that shed asynchronously, therefore, provide the best framework for differentiating among the thermal landscape, thermal efficiency, predator avoidance, and reproductive facilitation hypotheses. We tested the predictions above (and summarized in Table 1) in a population of asynchronously shedding prairie rattlesnakes ( *Crotalus viridis* ) in central Wyoming.

**TABLE 1:** An overview of the predictions we tested, generated from four hypotheses proposed explain aggregation during ecdysis.

Patterns predicted	Hypotheses			
	H1: Thermal landscape	H2: Thermal efficiency	H3: Predator avoidance	H4: Reproductive facilitation
Aggregation timing	Clustered around ingress and egress	Clustered around ingress and egress	Only during ecdysis	Only during mating
Sex of aggregated individuals	More gravid females	More gravid females	No strong prediction	At least 1 male and 1 female
Age of aggregated individuals	Fewer juveniles	More juveniles	No strong prediction	Only adults

## 2 Materials and Methods

### 2.1 Species Description

The prairie rattlesnake is a large‐bodied pit viper whose range extends from Alberta and Saskatchewan through northern Mexico. Individuals from the population we studied are active from mid‐May until early October. This population does not appear to aggregate overwinter, nor during gestation or parturition. The reproductive phenology of this population is unknown, but we witnessed one instance of copulation in late summer. Based on the phenology of other rattlesnakes, mating could occur in late summer only or in both spring and summer (Clark et al. 2014).

### 2.2 Study Site

We collected data from a high‐elevation (1800–2400 m asl) population of prairie rattlesnakes in west‐central Wyoming from April 2024–October 2025. The study area is a canyon with a road and creek bed at the bottom that run from east to west, separating two markedly different habitats. The south‐facing slope is dominated by abundant, rocky cover and deep fissures, which are used by prairie rattlesnakes to overwinter and during the active season. Canopy cover on the south‐facing slope is sparse, with small patches of sagebrush (primarily *Artemisia* spp.) and forbs (primarily *Euphorbia esula* ). At the canyon bottom, deciduous trees and grasses are more abundant, though rattlesnakes do not appear in these habitats until late spring and summer. The canyon's north‐facing slope is densely populated by conifers and minimal mid‐story cover. Rattlesnakes are present in the forested areas during the active season and use large talus fields embedded in the forest to overwinter.

### 2.3 Data Collection

We captured rattlesnakes found during visual encounter searches or while locating other VHF‐marked individuals. We color‐marked all captured snakes using non‐toxic acrylic paint (injected into the rattle) for individual identification at a distance and to prevent double counting. For 29 rattlesnakes of suitable size and health, we also implanted VHF transmitters (HOLOHIL SI‐2 or SB‐2) into the coelomic cavity under general anesthesia following methods described in Reinert and Cundall 1982 with improvements recommended by Hale et al. 2017. We recorded snout‐vent length (SVL), mass, sex, age class (juvenile or adult), and reproductive status (male, pregnant female, nonpregnant female) for each rattlesnake during its initial capture. We classified female rattlesnakes as adults at 651 mm (the smallest female with ovarian follicles found at this study site) and males as adults at 535 mm (the smallest mature male *C. oreganus* found in Macartney et al. 1990). We captured and collected morphometric data for each individual no more than twice per active season.

We tracked VHF‐marked rattlesnakes daily, although we only sought to obtain a visual record approximately once weekly to limit disturbance. When we observed a snake, we recorded group status, group size, and shedding status. We considered a rattlesnake to be in a group if two or more animals were observed in visual contact with one another (generally < 1 m apart). Snakes with known locations that could not be seen (ex: VHF‐marked individuals under a rock) were excluded from the analysis because we could not confirm group size nor shed status. We defined a snake as undergoing ecdysis if it had cloudy spectacles and/or visibly peeling skin.

### 2.4 Data Analysis

To determine whether ecdysis was a predictor of aggregation, we constructed a binomial mixed‐effects model in R:

$$\text{Aggregated}\;\left(y/n\right)\sim \text{Shed status}\;\left(y/n\right)+\left(1\;|\;\text{Individual}\;\mathrm{ID}\right)$$

We examined these data for temporal autocorrelation using DHARMa scaled residuals with a simulation‐based test of independence between residuals and time in R. We detected autocorrelation in 2 individuals, but our results were robust to their inclusion.

We used a Fisher's exact test to test for sex‐based grouping within aggregations.

## 3 Results

We included data from 81 individuals of all sex and age classes, 29 with VHF telemetry implants and 52 that we encountered opportunistically during telemetry or visual searches. We collected data from April 2024–October 2025. We determined that a rattlesnake's shed status was highly associated with the probability that it was in an aggregation, with an odds ratio of 23.72 (95% CI: 3.22–174.65, *p* < 0.001), and half (*n* = 12) of all aggregations contained a rattlesnake undergoing ecdysis. These results suggest that ecdysis and aggregation are spatially and temporally linked in this population.

### 3.1 Timing of Ecdysis and Aggregation

**FIGURE 1:**
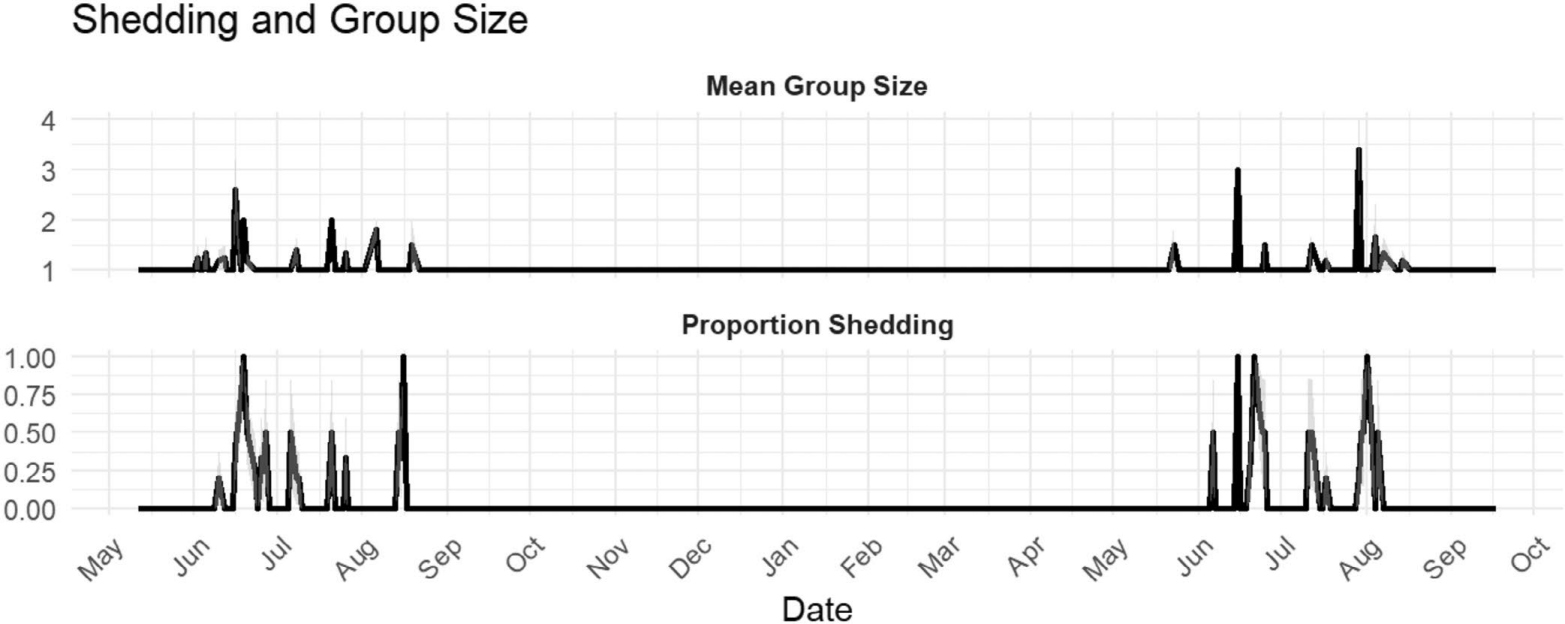
Mean group size and proportion of detected rattlesnakes that were undergoing ecdysis from April 2024–October 2025.

Ecdysis occurs from June until August (Figure 1). We observed 26 ecdysis events from 25 individuals during the study period (one individual was observed in both 2024 and 2025). Most individuals were observed only once or twice during the short shedding period, and 19 were never found in an aggregation. We collected data on 24 aggregations of prairie rattlesnakes, which were detected from May–August. We detected no aggregations during September and October. We observed 2 individuals in most aggregations, though we also captured 2 groups of 3 snakes and 2 groups of 4 snakes (range = 2–4 individuals, x̄ = 2.45).

### 3.2 Sex Composition and Reproduction

We detected both sexes in over half of the 24 aggregations (54.2%, *n* = 13). Of the single sex aggregations, we detected only males in 1, and only females in the remainder (41.7%, *n* = 10). The proportion of each sex class observed did not differ within aggregations compared to snakes captured singly (Fisher's exact test, *p* = 0.97, Cramér's *V* = 0.0853). We observed copulation once (on 6 August 2024) and neither individual was undergoing ecdysis during that encounter.

### 3.3 Age Class Composition

We observed one small snake in an aggregation during spring emergence in 2025, but we were unable to capture the individual to confirm whether it was a juvenile or adult. We did not detect any post‐natal aggregations nor aggregations of mothers with neonates.

## 4 Discussion

We believe our results are the first documentation of aggregation during ecdysis in prairie rattlesnakes. We detected 24 aggregations of 2–4 snakes each, 50% of which contained an individual undergoing ecdysis. We proposed 4 hypotheses for the underlying cause of shedding aggregations in prairie rattlesnakes and we found some support for the reproductive facilitation hypothesis and the thermal landscape hypothesis, whereas our results did not support predictions of the thermal inertia hypothesis. Our results were equivocal in regards to the predator avoidance hypothesis.

### 4.1 Thermal Landscape

The thermal landscape hypothesis states that rattlesnakes congregate in habitats that allow them to optimize thermoregulation during ecdysis. Under this hypothesis, aggregation itself does not confer a selective advantage. Rather, individuals simply coexist in the same high‐quality habitat during periods of increased metabolic demand or thermal constraint. This type of thermally induced habitat limitation has been proposed as a mechanism for other rattlesnake aggregations including those at overwintering (Bruckerhoff et al. 2021; Gregory 1984) and rookery sites (Moniz et al. 2024). We found no evidence that individuals in this population overwinter communally or attend rookery sites. This is unusual at such a high latitude, high elevation site, where thermal constraints on habitat use are strongest (Powers et al. 2025). The physiological demands imposed by overwintering and pregnancy differ from those imposed by ecdysis. Yet, the lack of aggregation in these contexts suggests that high‐quality thermal habitat could be abundant at this site relative to the number and distribution of rattlesnakes (Harvey and Weatherhead 2006).

The thermal landscape hypothesis predicts that aggregations should be most common when thermal constraints are high (i.e., during the coldest part of the active season), rather than associated with ecdysis per se. Our findings do not support this: instead, only one aggregation was detected during spring emergence, and none in the months leading up to immergence. This finding could reflect reality or a limitation of our study: rattlesnake detection probabilities are positively correlated with substrate and air temperatures (Thacker et al. 2023). Though rattlesnakes maintain similar internal body temperatures regardless of sex class, pregnant females may be more constrained to habitats with a high degree of thermal stability (Moniz et al. 2024). Thus, the thermal landscape hypothesis predicts that pregnant females should be over‐represented within aggregations. Our results do not support this prediction, however, our sample size is small. The thermal landscape hypothesis makes additional predictions that this study did not test. Notably, it predicts a high degree of shedding site fidelity, and/or re‐use of previous communal shedding sites across years due to site limitation. This pattern has been observed in *C. oreganus* (Loughran et al. 2015), *C. concolor* (Parker and Anderson 2007), and *Agkistrodon conanti* (Lillywhite and Sheehy 2016). Though our study only covered two active seasons, none of the communal shedding locations appear to have been re‐used, although we conducted regular searches at each. More prolonged observation could determine whether individuals in this population re‐use communal shedding sites.

### 4.2 Thermal Efficiency

The thermal efficiency hypothesis states that rattlesnakes aggregate to increase their thermal inertia via proximity to other rattlesnakes. Like the thermal landscape hypothesis, this hypothesis centers around the thermal constraints imposed by ecdysis. By contrast, however, aggregated individuals under this hypothesis have a selective advantage conferred by other snakes rather than by the environment. This hypothesis does not necessarily assume habitat limitation but has been implicated in scenarios where habitat limitation is also present (ex: newborn *C. cerastes;* Reiserer et al. 2007). An ectotherm's thermal inertia is inversely proportional to its surface‐area‐to‐volume ratio and describes the rate of cooling or heating of an animal relative to its environment (Christian et al. 2006). High thermal inertia permits individuals to maintain more stable body temperatures while minimizing time spent thermoregulating (Graves and Duvall 1987). By aggregating, rattlesnakes effectively alter their surface‐area‐to‐volume ratio and increase their collective thermal inertia (Reiserer et al. 2007). This is particularly useful during ecdysis, when individuals are more sensitive to temperature variability (Hoffman et al. 2021). The benefits of aggregation—from the perspective of thermal inertia—vary across multiple temporal scales, though we tested predictions derived from seasonal variation only. In this population, aggregation should be most beneficial during immergence and emergence, when the rate of heat loss to the environment is highest. Our findings do not support this prediction, however, as discussed above. Pregnant female rattlesnakes may prioritize a stable thermal environment more than nonpregnant or male rattlesnakes do (Moniz et al. 2024) and may therefore be over‐represented in aggregations. Our observations do not support this prediction.

**FIGURE 2:**
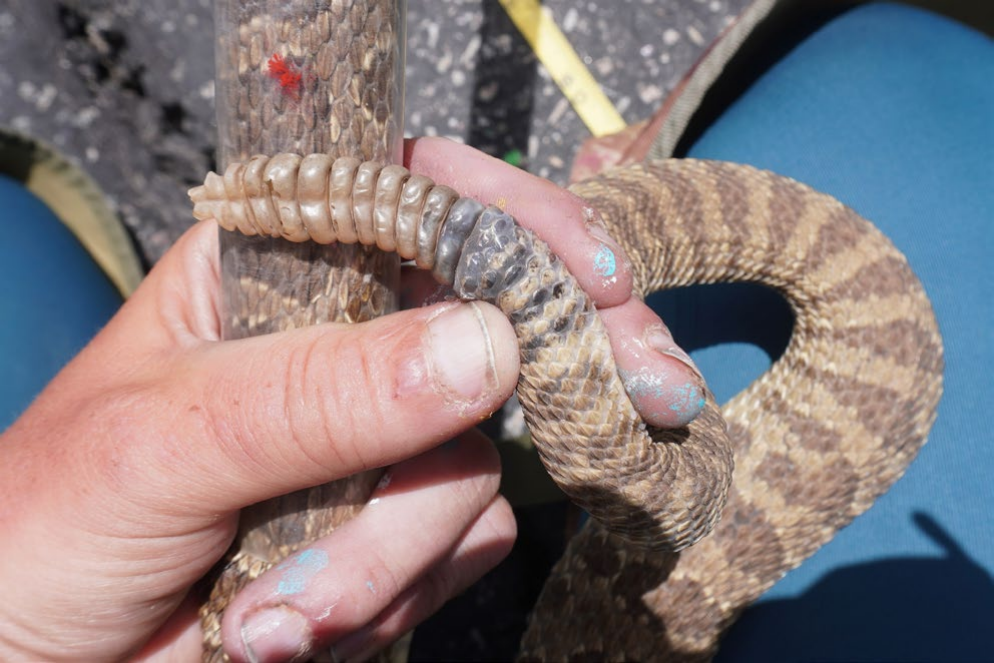
A prairie rattlesnake entering pre‐ecdysis. Note the newest proximal segment, which is still partially covered by skin.

The thermal efficiency hypothesis also predicts that juvenile rattlesnakes—which have higher cooling and heating rates than adults—should be overrepresented in aggregations (Loughran et al. 2025). Our findings do not support this overall, although we observed a possible juvenile within an aggregation during emergence. Interestingly, despite placing several cameras and following 8 VHF‐marked pregnant females, we observed no aggregations of neonates nor of neonates with adult females in this population. Post‐natal aggregations are documented in prairie rattlesnakes (Holycross and Fawcett 2002) and have been observed at neighboring sites. This finding may indicate a stable thermal environment that permits offspring to disperse immediately after parturition (Reiserer et al. 2007) or it may reflect low rates of detection for neonates overall, due to more cryptic behavior and small body size. Thermal inertia can be estimated more accurately using measurements of body temperature and heat loss over time (Dzialowski and O'Connor 2001). Further investigation of this hypothesis could use implanted temperature loggers to construct more appropriate models of thermal inertia rather than testing predictions based on differences among demographic classes like age or sex. An additional consideration for both the thermal inertia and thermal landscape hypotheses is that the thermoregulatory demands of ecdysis may be most pronounced in the pre‐ecdysis phase, when cell proliferation is highest (Gibson et al. 2011). In our study, individual color variation and limited visual observations made it impossible to reliably classify animals in pre‐ecdysis, except when the rattle was visible and the deposition of a new segment could be confirmed (Figure 2). Future research could use a more hands‐on approach to test the prediction that individuals undergoing pre‐ecdysis should be overrepresented in aggregations under these hypotheses.

### 4.3 Predator Avoidance

The predator avoidance hypothesis states that rattlesnakes aggregate to compensate for reduced visual acuity and reduced capacity to defend against predation during ecdysis. Several mechanisms have been proposed to explain the role of aggregation in reducing predation risk: being grouped may dilute individual risk, permit improved threat detection, or confuse predators (Lehtonen and Jaatinen 2016). Because this hypothesis directly implicates ecdysis as the cause of heightened risk, it also predicts that rattlesnakes should aggregate during the later stages of ecdysis (i.e., when their eye caps are cloudy) only. Therefore, aggregation behavior should be associated with ecdysis rather than temperature or environment. Though we had insufficient data to test whether ecdysis and aggregation behavior covary, we found that ecdysis increased the probability of an individual being found in an aggregation. The predator avoidance hypothesis makes no strong prediction about the sex classes present within aggregations. Generally among snakes, mate‐searching and movement behavior of males can increase their encounter rates with predators (Noble et al. 2025). However, this pattern is not ubiquitous. Pregnant viviparous reptiles could be more vulnerable to predation during gestation due to differences in basking behavior (Olson et al. 2015). Given the variable nature of the relationship between predation and sex class, our results are not conclusive regarding the predator avoidance hypothesis. Similarly, although patterns of mortality in juvenile snakes suggest that predation risk inversely scales with body size (Bonnet et al. 1999), it is difficult to study predation in snakes directly. Thus, this hypothesis makes no strong prediction about the age (which we use as a proxy for size) of individuals present within aggregations. Gathering more information about relative predation risk would permit us to make stronger, directional predictions to evaluate this hypothesis.

### 4.4 Reproductive Facilitation

The reproductive facilitation hypothesis states that rattlesnakes aggregate during ecdysis to facilitate successful reproduction (e.g., to permit mate‐guarding by males or to capitalize on a short breeding window following ecdysis) (Duvall et al. 1992). This hypothesis predicts, therefore, that aggregation will be associated with copulation rather than ecdysis per se. We have no information about the reproductive cycle of this population except for a copulation witnessed in late summer 2024. However, prairie rattlesnakes typically reproduce seasonally, and copulation in other Wyoming populations occurs from early July until late August (Duvall and Schuett 1997). Thus, this prediction does not seem supported by the timing of aggregations that we observed. The reproductive facilitation hypothesis predicts that all sexes should be present within aggregations, but that each aggregation should contain at least one of each sex. Only about half of the aggregations we observed were confirmed to contain at least one male and one female, which does not support this prediction. However, it is not always possible to capture or observe all individuals present within aggregations. The reproductive facilitation hypothesis also predicts that only adults (i.e., individuals capable of reproduction) should be present within aggregations. Our confirmed results support this prediction, although we observed a small snake (a small adult or large juvenile) in an aggregation during emergence in 2025.

### 4.5 Summary

Our results support a non‐incidental association between aggregation behavior and ecdysis in this high‐latitude, high‐elevation population of prairie rattlesnakes. We found some support for the reproductive facilitation and thermal landscape hypotheses as the adaptive purpose of shedding aggregations in this population and found no support for the thermal efficiency hypothesis. Roughly half of the aggregations we detected contained a male and at least one female, while the other half contained pregnant females, except for one male–male pair. These two seemingly distinct types of aggregation could suggest that shedding aggregations serve multiple purposes in this population. We have proposed several additional predictions that could be used to ascertain the relative influence of each mechanism with more certainty. The social behavior of squamates can be cryptic and difficult to observe directly (Clark et al. 2012). Understanding the mechanisms that underlie behavioral variation in response to physiological challenges can therefore provide valuable insight into poorly understood patterns of variation and improve our understanding of these taxa. Moreover, we can learn more about the function of this cryptic behavior if field biologists routinely record whether snakes are aggregated or shedding during each encounter.

## Author Contributions

**Emily Martin:** conceptualization (lead), data curation (lead), formal analysis (lead), funding acquisition (supporting), investigation (lead), methodology (lead), software (lead), validation (lead), visualization (lead), writing – original draft (lead). **Courtney J. Conway:** funding acquisition (lead), project administration (lead), supervision (lead), writing – original draft (supporting), writing – review and editing (lead).

## Funding

This work was supported by the Jackson Fork Ranch and Joe Ricketts, the National Science Foundation's Graduate Research Fellowship Program, Save the Snakes, and the Idaho Chapter of The Wildlife Society.

## Conflicts of Interest

The authors declare no conflicts of interest.

## Data Availability

The data and code that were used for these analyzes are provided in the Supporting Information.
